# Singular Case of Monstrosity

**Published:** 1859-01

**Authors:** S. C. Gholson

**Affiliations:** Holly Springs, Miss.


[Correspondence of the Cincinnati Lancet and Observer.]

### *Singular Case of Monstrosity.*

HOLLY SPRINGS, Miss., Nov. 29, 1858.

EDITORS LANCET AND OBSERVER,

*Dear Sirs:*—It was my good luck (or misfortune?) to meet with a short time back, an instance of (for want of a better name) "Teratogeny"—an exaggerated sort of "Xiphopages," that I consider worthy of a brief notice and a few reflections. Examples of this kind are rare, certainly, and, I confess, to me unprecedented, for I am unable, now, to recall, in any of the museums I have visited in this country or Europe, a case analogous. I was requested to visit, professionally, a negro woman, who, her master informed me, had symptoms of "miscarriage." I found her morose and disinclined to the communicativeness so usual with her "race." She is well developed, of good figure, stout and healthy, of that color or complexion denominated here "dark chocolate," being a few degrees removed from *noir foncé* of the pure blooded African To the common interrogatories, in reference to her previous condition and the character and frequency of pains, etc., etc., I with difficulty extracted, substantially, that she was in the twenty-fifth year of her age, and had given birth, in as many labors, to three living and well formed children, perfect after their kinds, black or mulatto; that she was six months' "gone," and had experienced, for some time, sensations attributable to "quickening;" that the pains had been quite frequent and of regular recurrence; that the membranes were ruptured, and the liquor amnii "par conséquence," discharged. I afterwards, however, became convinced that she was in error in regard to the stage of gestation arrived at. Such errors are common. She had met with no accident, and experienced no violence, and the only cause assigned for the apprehended untoward termination of gestation was the excitement occasioned by the sudden death of one of her children.

Having found her lying face downwards, the thighs flexed upon the pelvis, I ordered her to turn upon her back, that I might make the necessary examinations. Instead of obeying, however, she got up quickly, sat a few moments upon a chair, shook her dress, and return-to bed. I was not surprised to find the contents of the womb upon the floor, and a glance sufficed to inform me that I had a specimen, at once rare and interesting, of gemellary conception.

Imagine (and excuse the implied duality) *two* diminutive creatures of the "*genus homo*" united, not by a musculo-fibrous medium, but soldered, if the expression be admissible, and inseparably fused into one, from the manubrium sterni, or where that should be, to a common umbilicus, so that, if there be no vertical diaphragm separating the thoracic and abdominal cavities of these "freaks," those cavities must be common to the double fœtus. There is but one navel, one umbilical cord, and one placenta. The umbilicus occupies a central position in the "fourchette," separating the hypogastric regions and pelvis of the fœtus (I use the twin in in the plural), and offers nothing unusual in its appearance. The chord is small, delicate, and apparently—for I made no dissection, to preserve the specimen entire—contains but one set of vessels. The placenta is large, vascular, and seemingly well suited to its double functions, and I may here say that, although the expulsion was not effected in involucra, the placenta was extruded simultaneously with the fœtus, or at any rate the cord was left unbroken. In general aspect there is a wonderful and striking resemblance between the "twin sisters," for I ascertained their sex to be feminine, the external genitals being well defined, the labia and clitorides easily distinguishable. The period of gestation reached by these twins was, apparently, the end of the fourth or beginning of the fifth month (v. "*Cazeaux Traitè des Accouchement*"), yet are the features of both well marked and distinct: the arms, hands, and fingures, the legs, feet, and toes well proportioned and symmetrical, except where a superabundance of dermoid tissue, or a relaxed condition of the subcutaneous areolar structure mars the symmetry and regular contour of outlines, by permitting the skin to hang in folds in certain localities, as the neck, etc. The eyes are well seen through the separated palpebræ, and the tongues in the patulent mouths. The ears are small, but well formed and placed. The ani are also well represented by two unoccluded orifices *in situ proprio*; in fact, complete duality is maintained as to all the peripheral organs and members, the umbilicus excepted.

The mode of union between these little anomalies is not adventitions or intimediary, but there is, in appearance, a loss of substance or default in each, to render the fusion more perfect; in *aliis verbis*, the thoraces and epigastric regions of each are, to speak geometrically, truncated, to make the union more intimate. Indeed, Salmaces, so facetiously mentioned by the venerable Dr. Meigs, in an article of his on double monsters, would, I doubt not soon have wearied of so close a union with her lover, as exists between my little twins.

It is not my wish, nor is it my intention, to enter the broad field of teratological discussion; yet I cannot forbear a few of the many reflections to which the sight of this *lusus matronæ naturæ* has given rise. For the sake of simplicity, these reflections may be enumerated as obstetrical, anatomical, physiological, etiological, medico-legal, psychological, social, economical, and comical. I am aware that I would soon be over head and ears if I attempted to penetrate, didactically, into each of these divisions; I shall therefore only endeavor to do what our fine-witted friends over the pond would call *effleurer la matière!*

We cannot deny that the full term of gestation might have been arrived at by these twins, since nutrition having been maintained to the fourth month might well have progressed to the ninth, without accidental interference or violence. Supposing that period (the ninth month) to have been attained, would parturition be possible, under the circumstances, without sacrificing the mother or her offspring? I cannot conceive of its possibility, *per vias naturales*, by any variety of presentation, defined or imagined, without giving extraordinary amplitude to the pelvic-straits, or unusual diminutiveness to the double fœtus. The celebrated Siamese twins cannot be referred to as a precedent, since the union between them is less, very much less, intimate than in the present case, thereby permitting them a greater freedom of motion, laterally, to each individually, and the separation of one from the other by a considerable interval—circumstances that must have greatly facilitated them *in transitu ad auras*. The accoucher would therefore be reduced to two alternatives, provided he could make a correct diagnosis at all: the Cæsarian operation, attended with great peril to the mother and child, or the sectio-fœtus, of necessity fatal to the infant. Without further amplification on this point, let us pass to a few anatomical considerations.

The *point d' union* between these "freaks" presents no apperance of cicatrix, the integuments being reflected from one to the other. I have already explained that the connection between them is not intermediary, but, apparently, by an elliptical default, extending from the upperpart of the manubrium sterni to the hypogastrium—the long diameter of the ellipse being vertical, and the short diameter occupying the plane of contact of the twins in a transverse direction. I have used the word "apparently," because it is impossible to determine, without dissection, whether the sternum and sternal portions of the ribs, or their rudimentary representatives, exists in either or each fœtus or whether the union is entirely tegumentary, the ordinary anatomical characteristics being unaffected. I will also reiterate, here, the striking resemblance they bear each other, and the remarkable distinctness and perfection of the *visible* organs and members. The fact of there being but one umbilicus, and one cord, containing "apparently" but one set of vessels, must, under the division of the artery and venous tunics, be exceedingly interesting to the anatomist, for we can readily imagine the impossibility of a single set of vessels supplying this double economy. In all other points, subject to observation, complete duality seems to have obtained. The arrangement of the internal organs and of the vessels beyond the point of penetration must remain a matter of conjecture, as I have determined to keep the specimen intact.

Physiologically considered, it will strike the most casual observer how utterally at variance with the ordinary catalogue of physiological acts must have been the conduct of these unfortunate "*individuals*;" can I say?—could they have attained to a mundane existence. I need only cite the act of progression, which could never have been performed by both simultaneously in the usual direct way; one or the other would be forced to "perpetrate the paradox of *advancing backwards* to an object, or they would be compelled to approach sideways, or by waltzing, since, from their intimate union, they must have maintained a perpetual *vis á vis* to each other. I might mention numerous other instances of entire incompatibility with common physiology, but deem it an unnecessary consumption of time and a useless prodigality of space, since these unavoidable discrepancies will be apparent to every one.

The cause of this curious anomaly must, I fear, ever remain obscure. The influence of the maternal imagination, the accidental changes experienced by the fœtus during intra-uterine life, and a radical defect in the germs, are enumerated as the most probable causes of monstrosity. In the first place, "*me judice*," the imagination had no causative influence in this case; secondly, the anomalous union is too perfect to be the result of accident; thirdly, though a bad egg may spoil one "pudding," I can not conceive of its uniting two. In fact, such an array of causes amounts to the telling us, we must be satisfied with inserting this in the great dictionary of etiological obscurities, and wait with patience, until nature's God sees fit, if He ever does, to write after it the definition. The only at all plausible hypothesis, in the present case, is, I think, that pertaining to a double ovum or germ; but "Alabama"—"here we rest."

Let us make once more the almost, I will admit, absurd supposition, that these creatures had reached a separate, I mean extra-uterine existence; that they had attained an age to be affected by the social compact—consisting of certain concessions for certain rights and privileges, guaranteed, protected and insured by laws, rules and regulations: would they be amenable to the penal or other code that now governs society? Would they be considered as individuals acting in concert? Might not one commit a felony, the other being ignorant? and thereby render it impossible to punish the guilty, without inflicting an undeserved share of the penalty upon the innocent?—or, would each be considered as accessory, in every case, to the acts of the other, and, in consequence, be made legally responsible? Could twins, be construed as "*individuals?*" Could they act as "*individuals,*" and thereby come under the general law in reference to monsters? "Monsters, if capable of acting as individuals, have the same rights as other persons." I am free to admit, that I am unable to respond satisfactorily, and call upon medico-legalists and learned Doctors of Law to aid me in this dilemma; meanwhile, I will pass from Scylla to Charybdis—from medical jurispudence to psychology.

The psychological division of my reflections reduces me to the necessity of weighing each thought, and measuring each word. Like the befogged mariner, I must fell my way, or, what is better, cast anchor at once, to avoid the unseen breakers and quicksands—the rocky shoals of doubt and perplexity, upon which the unwary sailor might be enticed by the mirage of specious reasoning, or driven by the *flatus* of false argument. Stand fast, good cable, and part not, whist I exclaim, how rich a theme for the psychologist! What "piquant salad," what savory "cud" for the pensive jaws of some solitary Plato! Who shall say whether double souls exist? Who shall decide as to the condition of thse *animæ junctæ* in "the land of the hereafter?" What is the moral result of such physical "duplicity" as our little "freaks" would present? I appeal to psychologists, and to you whose work it it is to guide erring souls to a peaceful haven. Should you disagree, in the words of the Roman bard, "*non nostram tantas componere lites.*" A preaching doctor, like a praying horse, is liable to stumble: so, I will rest satisfied with murdering half a line, for the "English Homer," "whatever *will be, will be* right."

In a social, economical and comical point of view, I suggest that "Doesticks" and his "bussum friend, Damphool," make an experimental voyage of discovery, with my little hyper-Chang & Eng; and thinking it better to practice than to preach economy, I take advantage of this lumping conclusion, to invite the Faculty to visit "*mes*

*petits enfans*" at my office, where it will give me pleasure to exhibit them to the fraternity, without demanding "quarter" at the door.

In fine, I hope, sirs, that you wil not bring in a diagnosis of *cac. scrib.* against me for the length of this, but attribute it to the interesting nature of my "case of monstrosity.

S. C. GHOLSON, M. D.

